# Leadership, adaptation, and group resilience: a qualitative study of Bulgaria’s 33rd Antarctic summer campaign

**DOI:** 10.3389/fpsyg.2026.1820497

**Published:** 2026-04-24

**Authors:** Martin Milanov, Ivo Vlaev, Maria Stoicheva, Yavor Paunov

**Affiliations:** 1 Department of African Studies, Faculty of Classical and Modern Philology, Sofia University “Saint Kliment Ohridski”, Sofia, Bulgaria; 2 Centre for Behavioural and Implementation Science Interventions (BISI), NUS Yong Loo Lin School of Medicine, Singapore, Singapore; 3 European Studies Department, Philosophy Faculty, Sofia University “Saint Kliment Ohridski”, Sofia, Bulgaria; 4 Department of Economic and Consumer Psychology, Faculty of Social Sciences, University of Mannheim, Mannheim, Germany

**Keywords:** adaptation, Antarctica, expedition, isolation, leadership, motivation, psychology, team cohesion

## Abstract

**Introduction:**

Antarctic field expeditions are isolated, confined, and extreme settings in which specialists must rapidly form effective teams to protect their wellbeing and to accomplish mission goals. We investigated leadership, team cohesion, group identity, and group-level motivation and coping during Bulgaria’s 33rd Antarctic Summer Campaign using a longitudinal qualitative design.

**Methods:**

Semi-structured interviews were conducted before departure (*n* = 28) and after return (*n* = 36), complemented by on-site ethnographic observation and field notes from an embedded observer. Data were analysed using reflexive thematic analysis, contrasting group-level anticipatory narratives with group-level retrospective accounts rather than tracking individual change over time.

**Results:**

Participants described a marked transition from initial subgrouping (e.g., experienced vs. novice; scientific vs. logistics roles) toward a superordinate “one-station” identity. Leadership practices that emphasised transparent communication, mixed-group coordination, and active norm-setting were described by participants as supporting reduced friction and sustained collaboration. Motivation was generally high across the mission, with brief weather- and monotony-related dips that were buffered by humour, peer encouragement, and structured daily routines.

**Discussion:**

The findings highlight how shared purpose, interdependence, and identity-related leadership behaviours were perceived as supporting cohesion and adaptive coping in short-term polar campaigns.

## Introduction

Polar expeditions offer a unique context for natural and social scientists interested in human behaviour under extreme conditions. Antarctica is increasingly recognised as a compelling setting for psychological and anthropological research as human presence on the continent increases (O’Reilly, 2017; Schweitzer, 2017). The environment of Antarctic stations—isolated, confined, and extreme (ICE)—poses unique challenges to individuals and groups, making team dynamics and adaptation critical for both individual wellbeing and mission success (Palinkas, 2003; Palinkas and Suedfeld, 2008). Studies on Antarctic research stations have documented a wide range of psychological and social effects on personnel during missions: from positive experiences of camaraderie and personal growth to stress, interpersonal conflict, and mood disturbances (Palinkas and Suedfeld, 2008; Suedfeld and Steel, 2000). Previous research has also noted recurrent detrimental patterns such as the “third-quarter phenomenon”—a dip in morale at the mid-point of isolation missions (Bechtel and Berning, 1991; Palinkas and Suedfeld, 2008). At the same time, polar field campaigns often yield reports of strong group bonds and resilience, leading some to question the traditionally “pathological” narrative of life in extreme environments (Suedfeld and Steel, 2000; Palinkas, 2003). In such cases, the importance of motivation, coping, leadership, and team cohesion is often emphasised (Suedfeld, 1991; Palinkas, 2003). Therefore, understanding the interplay of these factors is both academically and practically relevant for expedition planners who must select and prepare individuals for effective teamwork in austere settings (Palinkas and Suedfeld, 2008; Marques-Quinteiro et al., 2024).

Motivation in Antarctic expeditions combines intrinsic drivers (curiosity, mastery, achievement) with extrinsic concerns (career development, national pride, adventure) (Mehta and Chugh, 2011; Palinkas and Suedfeld, 2008). Motivation trajectories are dynamic and often decline during routine phases and mid-mission periods. Coping—the mechanism converting stress into adaptation—employs problem-focused strategies (planning, task substitution), emotion-focused tactics (humour, reframing), and social approaches (support-seeking, communal activities; Tortello et al., 2021). However, coping effectiveness depends on context: cohesive team climates, fair workload distribution, and supportive leadership reduce avoidance coping and buffer motivational declines (Landon et al., 2015; Palinkas and Suedfeld, 2008).

In Antarctic teams, effective leadership can help specialists function as adaptive units by setting clear priorities, monitoring fatigue, communicating transparently, and fostering inclusion (Palinkas, 2003; Hannah et al., 2009). These leadership behaviours strengthen cohesion—defined as shared commitment and trust supporting mutual monitoring and help-seeking—through shared mental models, psychological safety, and role clarity (Golden et al., 2018). Cohesive climates with transparent communication and equitable workload sharing have been associated with lower avoidant coping and buffered motivational dips, whereas fragmented subgroups and ambiguous norms increase friction and error risk (Landon et al., 2015; Golden et al., 2018). Cohesion requires continual maintenance and mediates the impact of leadership on safety and performance, particularly when leaders promote a shared identity that links daily work to collective purpose (Haslam et al., 2011; Hogg, 2001).

Identity perspectives explain how leadership and group dynamics acquire motivational force in Antarctic microsocieties. The social identity approach posits that leaders are most influential when perceived as prototypically ingroup members and when they create and embed a shared sense of “us” (Hogg, 2001; Haslam et al., 2011). In Antarctic stations, an inclusive “one-station” identity reframes subgroup boundaries as complementary expertise within a single task system, increasing norm clarity, trust, and mutual support. Identity leadership thereby stabilises collective efficacy and buffers motivation fluctuations by aligning individual goals with the collective mission. Conversely, when leaders act in identity-incongruent ways (e.g., favoring a subgroup), the shared identity fragments; cohesion weakens, role conflicts rise, and coping shifts toward maladaptive patterns that undermine performance (Golden et al., 2018; Landon et al., 2015). Identity thus functions as a critical mechanism through which leadership may shape coping and perceived mission success in Antarctica.

This study examines how a Bulgarian Antarctic expedition team—comprising scientists, engineers, medical staff, military/logistics personnel, and a media documentarian—navigated the challenges of living and working together in Antarctica. Through self-reported and observational data, we investigated processes and behaviours perceived as critical for mission success: leadership, group cohesion and identity, group-level motivation trajectories, and coping mechanisms. We employed a qualitative approach combining open-ended interviews and participant observation to explore psychological and behavioural phenomena difficult to quantify—such as team members’ sense of shared mission or how they managed motivational fluctuations. By triangulating pre-mission expectations with post-mission experiences and on-site observer records, we identified themes and patterns to inform future expeditions. The pre-post design captures change over time, revealing shifts in anticipatory narratives, coping strategies, and cohesion across expedition phases. Embedded observation provides ecological context through interaction patterns, routines, and nonverbal cues that are often missing from interviews, thereby serving as an independent validation of self-reported themes.

Drawing on this literature, the present study was guided by three research questions: RQ1: How do expedition members describe leadership behaviours and group processes that support or hinder cohesion and a shared ‘one-station’ identity during a short-term Antarctic campaign? In our interviews, ‘leadership behaviours’ referred to behaviours such as priority setting, communication, and inclusion, drawing on work on leadership as openness, accessibility, and availability (Heifetz et al., 2009; Yukl, 2013; Carmeli et al., 2010). ‘Cohesion’ and ‘identity’ referred to perceived shared commitment, trust, and a sense of ‘we’; RQ2: How do participants characterise their motivation trajectories and coping strategies across different phases of the mission at the group level? ‘Motivation’ and ‘coping’ here encompassed perceived energy, purpose, and strategies for handling stressors. RQ3: How do relations between different functional sub-teams (e.g., scientists, logistics, construction) evolve over the course of the expedition, and how are these dynamics perceived in retrospect?

## Materials and methods

### Participants and setting

The study involved members of the 33rd Bulgarian Antarctic Expedition (a summer campaign to Livingston Island lasting ~4.5 months). Twenty-eight (pre) and 36 (post) members participated in the study. Participants’ ages ranged from their late 20s to their early 60s (median age was 44 years). Member occupations varied, including scientists from the geosciences and life sciences, engineers and technicians, logistics personnel (including military officers responsible for transport and base operations), a medical doctor serving as team physician, a documentary filmmaker/journalist covering the mission, and several general support staff (electrician, mechanic, cook). Many had extensive prior expedition experience—about one-third were veteran Antarctic expeditioners who had been to the Bulgarian base multiple times—while others were first-timers. All participants were Bulgarian except for two foreign collaborating scientists (from Spain and Turkey) who joined part of the expedition. Individual deployment durations ranged from approximately 3–12 weeks, depending on team assignment (early vs. late rotation) and functional role.

The expedition was based at “St. Kliment Ohridski Station” on Livingston Island (South Shetland Islands) during the austral summer (December–March 2024/5). The base is relatively small and confined: it comprises a few main buildings (dormitories, laboratories, galley) and can accommodate no more than a few dosen people. During the study period, the station population fluctuated between 18 and 30, with some members arriving or departing in stages (typical for summer campaigns). The setting is characterised by environmental extremes—frequent high winds, rain and snow, cold temperatures, constant daylight in midsummer shifting to partial darkness by the end of the season—and complete physical isolation—the base is situated approximately 3,000 km from the nearest continental port. Communication with the outside world was available via satellite internet and radio, though bandwidth was limited.

### Procedure, design, and data collection

We employed a longitudinal qualitative design with two interview phases and continuous observation. Pre-expedition interviews were conducted in Aheloy, Bulgaria, 2 weeks before the first team departed for the mission and about 2 months before the last team’s departure to Antarctica. Each participant took part in semi-structured interviews (5–30 min) conducted in Bulgarian (the interviewers were bilingual researchers who later translated and transcribed relevant content into English). Pre-expedition interviews took place during a 2-day team-building meeting in Aheloy. They focused on anticipatory expectations regarding cohesion, motivation and inter-team relations. Post-expedition interviews were conducted after all teams returned to Bulgaria, typically between approximately two and 6 weeks after each participant’s return, reflecting the staggered departures and arrivals of the five mission teams. Since the interviews were anonymised, we could not calculate the exact time each interview was conducted before or after each participants departure/arrival. The shorter pre-expedition interviews typically contained multiple, complete responses to our core open-ended questions about cohesion, motivation, and inter-team relations, and we use them to characterise anticipatory frames at the group level rather than to draw fine-grained individual trajectories.

The interviews were conducted voluntarily, and participants were informed of the anonymity and purpose of the interviews. The collected data was saved securely on an encrypted drive and used only for the transcription of the recorded interviews. All participating members of the mission provided explicit consent to participate voluntarily.

The interviews covered three broad questions related to the upcoming mission:

1) “Which factors do you expect to be unifying across the different teams during the expedition?”—exploring perceptions of leadership, team cohesion and identity;
2) “How do you expect your levels of motivation to change during the mission?”—addressing motivation trajectories and potential high/low points;
3) “How do you expect the relations between the different teams (science, logistics, etc.) will change during the mission?”—anticipating any shifts in inter-group dynamics over time.

Two additional questions were related to different research outside of the scope of the present study and are omitted here.

An embedded observer accompanied the team from the start of their journey and was present on-site at the Antarctic base for approximately a month, providing important context and triangulation for the self-reported interview data. This observer kept detailed field notes throughout travel and station residency, focusing on interactions, behaviours, and events relevant to group dynamics, motivation, and adaptation. Daily logs were recorded and expanded into complete narrative entries whenever time allowed. Sample field notes documented the atmosphere during transit (e.g., how a sense of shared national pride visibly lifted the group’s spirits when others at airports asked about their mission), the formation of subgroups (e.g., an “experienced” vs. “novice” clustering observed in early days), conflict or stress incidents (e.g., a minor dispute among the construction crew over work allocation), and leadership actions (e.g., the base commander’s efforts to organie cross-team social activities). We foreground this novice–experienced axis because it emerged repeatedly in both interviews and field notes as a primary way in which participants themselves interpreted adaptation and cohesion—for example, contrasting ‘old hands’ who knew the routines with newcomers who were still learning the environment. Functional roles (e.g., scientist vs. logistics) often overlapped with experience level, which made the novice–experienced distinction particularly salient in participants’ narratives.

The observer was introduced to expedition members as an independent researcher studying team processes; they participated in daily life but did not intervene in decision-making. The observer’s presence thus covered roughly the second month of the on-station phase, providing contemporaneous but necessarily partial triangulation of self-reported patterns, especially with respect to early team formation and emerging routines.

After the expedition’s completion and return to Bulgaria, post-expedition interviews were conducted (some individuals from the pre-expedition sample were unavailable due to travel plans or other commitments; additionally, some participants took part in only one of the two interview waves). The post-expedition interview guide mirrored the pre-expedition one, asking the same five key questions, now phrased in the past tense. This symmetry facilitated comparison between pre-expedition expectations and post-expedition experiences at the thematic level. Each post-interview lasted 30–75 min. All interviews were audio-recorded, transcribed, and translated into English for analysis. To protect anonymity, names were removed or masked. Quotes are labeled using the interview question number (Q1–Q5), interview wave (PRE/POST), and an anonymised respondent identifier (e.g., “Q2PRE06”). Because interviews were anonymised, respondent identifiers are not linkable across the PRE and POST waves; thus, the pre–post comparisons reported in this article concern thematic patterns at the aggregate group level rather than within-person developmental change.

### Data analysis

Collected data were analysed using reflexive thematic analysis following Braun and Clarke (2006). Analysis proceeded iteratively through (1) familiarisation with the interview transcripts and the embedded observer’s field notes; (2) generation of initial codes; (3) development, review, and definition of candidate themes; and (4) write-up with selection of illustrative extracts. Coding followed a hybrid approach: deductive codes were informed by the interview guide and focal constructs, while inductive codes captured unanticipated patterns emerging from participants’ narratives. Observer field notes were coded in parallel and used to triangulate interview-based themes; illustrative alignments between observational statements and interview excerpts are shown in Table 1. Overall, post-expedition interviews tended to be longer and more detailed than pre-expedition ones, so they provided the bulk of illustrative material in the Results, while pre-expedition interviews mainly informed anticipatory frames at the group level. In a few cases, field notes and interview narratives diverged (e.g., early tensions later downplayed in retrospect), which we interpret as evidence of retrospective reframing rather than as errors in either source. Examples of codes and their grouping into themes are provided in Appendix 1, Table A. In most cases, the observer’s notes converged with participants’ later narratives for instance, both sources emphasised all-hands cooperation during demanding tasks and the emergence of a ‘one big family’ ethos. In a few instances, however, field notes recorded early tensions between sub-teams (e.g., over work allocation) that some participants subsequently downplayed or framed as minor in retrospect. We interpret such discrepancies as illustrating the reconstructive nature of post-hoc narratives, underscoring the value of treating interviews and observations as complementary, situated perspectives rather than as interchangeable records of an objective sequence of events. Deductive coding drew directly on our working definitions of leadership (e.g., priority-setting, communication, inclusion), cohesion and identity (e.g., perceived shared commitment, trust, ‘we-ness’), and motivation and coping (e.g., perceived energy, purpose, and stress-management strategies), which also informed the interview questions. This operationalisation is necessarily broad and perception-based, and we treat these constructs as situated interpretive categories rather than as precisely measured variables.

**Table 1:** Triangulation matrix: observer field notes aligned with interview excerpts.

Observer statement	Aligned interview quotes (shortened)
From the start, a clear division formed between ‘experienced’ and ‘new’; experienced bonded quickly, while novices had basic questions answered caringly to calm them.	- PRE01: Important to listen to experienced leaders who have been there before they know more. - PRE07: No idea, first time; no comparison base.
Groups form instantly based on prior ties, creating slight new/old divide; novices uncertain, ask more than informed.	- PRE07: No idea, first time; no comparison base. - POST23: Sleep/eat together constantly difficult to tolerate; built some distance.
Experienced take what they need and start independently; novices wander or beg to join.	- POST01: First-timers handle basics responsibly others take for granted without process experience. - POST23: Wait together daily best relations, but moments of not wanting to see them
Number of prior visits is a vain topic often mentioned; does not diminish novices’ work.	- POST01: First-timers responsible for basics like fuel/checks as vital as complex tasks; novices undervalue without experience.
Experienced vs. novices in bad weather: novices restless/questioning; experienced conserve energy knowing limits.	- PRE01: Listen to experienced leaders they know the environment. - POST23: Constant togetherness from childhood ties difficult to tolerate.

## Results

The analysis revealed a variety of themes related to team dynamics, motivation, and adaptation, with many participants’ post-expedition reflections closely mirroring—and in some cases starkly contrasting—their pre-expedition expectations. Overall, the expedition team reported high levels of cohesion and effectiveness, despite encountering challenges along the way. In this section, we first present an overview of key insights from the pre- and post-interviews, contrasting participants’ expectations before deployment with their experiences during the expedition. Because the post-expedition interviews were generally longer and more elaborated than the pre-expedition interviews, the following sections draw more extensively on post-expedition narratives when illustrating themes. Pre-expedition interviews primarily illuminate anticipatory frames, while the embedded observer’s field notes offer a contemporaneous point of comparison for these retrospective accounts. These patterns should be interpreted as aggregate, thematic trends rather than individual longitudinal trajectories because interviews could not be linked across waves.

### Overview of participants’ expectations vs. experiences

#### Team cohesion, leadership and identity

Pre-expedition expectations: The dominant expectation was that a common mission goal (building and maintaining the base, completing science projects) would be the primary unifying force. Many emphasised mutual reliance for survival and success: “If we do not help each other, nothing will get done” (mentioned multiple times across interviews). Leadership was expected to play a key role in synchronising teams and smoothing any frictions. Some participants also mentioned shared national pride and the spirit of adventure as potential bonding factors (Q2POST2, Q2POST26, Q2POST27; Q5PRE5,16).

Post-expedition experiences: A strong shared purpose was indeed confirmed: accomplishing the mission’s goals together was repeatedly cited as the “glue that held the team together”. Interdependence was experienced uniformly—*“you have no one else to rely on but each other* in such a remote place” (Q2POST1). A robust group identity developed a sense of “we became like family” (8 answers in the interviews contain comparison with family)^1^, often reinforced during challenges and triumphs. In total, we identified 31 answer segments that reference “family” and “mutual reliance/ interdependence”. Similarly, four interviews mention “…and everyone wants to help the other. *Just because there is no one else to turn to.”* (Q2PRE12). The observer noted that everyone “was in it together” from early on, with even newcomers quickly absorbing a norm of helping each other. However, some subgroup divisions did arise: for instance, a temporary rift between the logistics and science teams was noted when communication lapsed, though leadership interventions (regular all-hands meetings and mixed-group activities) helped reintegrate the teams, with these patterns also reflected in the triangulation matrix (Table 1).

#### Motivation

Pre-expedition expectations: Participants anticipated sustained motivation, often citing past toughness or professional passion (e.g., “I am used to tough environments”) (Q3PRE5). Some expected an energy boost from team dynamics, while a few cautiously acknowledged that weather or monotony might cause temporary declines in morale. Experienced personnel predicted minimal change based on prior expeditions.

Post-expedition experiences: Motivation remained high, primarily sustained by meaningful work and peer support. Mid-expedition weather and routine did cause brief enthusiasm lapses (one participant noted: “monotony does affect you after a while”) (Q3POST4), consistent with the “third-quarter” effect. However, no serious demotivation occurred. Motivation resurged near the mission’s end as the team pushed to “finish strong,” bolstered by the anticipation of return. Personal commitment to the mission and group camaraderie proved key to maintaining motivation through temporary dips. In longer missions, patterns of increased depression and insomnia in early winter have been observed during winter-over residencies (Kang et al., 2022).

#### Inter-team relations

Pre-expedition expectations: Most anticipated eventual cohesion across subgroups, with an initial “feeling-out period” expected to yield a unified team. Participants recognised potential tensions between different backgrounds (scientists, support staff, construction crew) but believed strong leadership and clear rules would prevent friction and clique formation. Some veterans cautioned that extreme individualists might resist group adaptation.

At the same time, functional roles did matter. For example, logistics and construction crews often shared work rhythms distinct from laboratory-based scientists, which initially reinforced subgroup boundaries and differing perceptions of workload. As mixed crews were increasingly formed and interdependence became salient, however, participants described these roles less as competing silos and more as complementary contributions to a single station-wide task system. We foregrounded the novice–experienced axis here because participants themselves repeatedly used experience level as their primary lens for interpreting adaptation and cohesion, while role differences, although present, were usually discussed through that experiential framing. We therefore report results primarily along the experienced–novice dimension, which participants themselves used as their main lens for making sense of adaptation and cohesion, while noting role-based differences (e.g., science vs. logistics) where these added distinct nuance beyond experience level.

Post-expedition experiences: High cohesion was achieved by mission’s end; nearly all participants reported becoming “one big team, regardless of roles” (Q2POST19). Early us-versus-them divisions, such as scientists clustering together or construction crews bonding separately, emerged initially but dissolved as members recognised mutual contributions and spent time working together. An early-weeks logistics-science team split, attributed to differing work rhythms rather than animosity, was bridged through leadership interventions (team meetings and mixed-task assignments). After approximately 1 month, most cliques dissolved into a cohesive community. By final weeks, mutual respect predominated, with members recognising interdependence despite not all becoming close friends. Overall, leadership’s deliberate efforts to integrate subgroups successfully met expectations for eventual unity.

### Thematic narratives and underlying factors

#### Group cohesion

“*We became like a family – everyone depended on everyone.*” (Q2POST10).

A dominant theme was the high degree of team cohesion that developed during the deployment, confirming most members’ pre-expedition belief that shared goals and mutual reliance would bind the group. From the outset, participants expected that survival and mission success in Antarctica would require intense cooperation: “If we do not help each other, nothing will get done” (Q2PRE06). Post-expedition, nearly all interviewees remarked that different sub-teams (scientists, engineers, logistics, etc.) “melted into one unit” throughout the expedition. They described an atmosphere of collectivism in which everyone was ready to assist one another, regardless of job description. The observer’s journal independently corroborated this all-hands cooperation, detailing how, even in off-hours, people would spontaneously join in tasks such as unloading supply crates or shovelling snow, reinforcing a norm of “we are all in this together.” It was essentially unthinkable at the base for someone to sit idle if others were struggling with a job; a norm quickly emerged that all available hands pitch in until the work is done before anyone rests. Participants commonly referred to the team as “one big family” or “one organism.” As a team member with prior experience put it, “Your whole existence and lifestyle are shared…everyone looks after everyone” (Q2PRE10).

#### Underlying factor: *common mission and purpose*

The overarching scientific and logistical goals unified the team before and during the expedition. Members were motivated by shared purpose—framed as idealistic (“we all have one goal: to make and maintain the base”) and reinforced through tangible accomplishments, including building a fuel storage facility, installing a weather station, and conducting a topographic survey (Q2POST1). These collaborative tasks fostered collective pride in contributing to a legacy, as exemplified when a participant exclaimed during cement pouring, “Years from now we will tell our grandchildren we poured the concrete for the Antarctic base!” This sense of meaningful contribution gave their work significance beyond immediate tasks. Notably, team members displayed this collective pride throughout the expedition—even to fellow travelers en route—embodying a shared identity as Bulgarian Antarctic explorers that became perhaps the most unifying factor early on, lifting morale across the group.

#### Underlying factor: *mutual dependency in extreme conditions*

Consistent with expectations, the harsh reality of Antarctica made it clear that everyone’s safety and wellbeing depended on others. Post-expedition interviews stressed this repeatedly. A young researcher (Q4PRE27) reflected: “In extreme conditions, people get much closer, because instinctively they know that their life depends on each other. Thus, the connection is powerful and transferable. That dependence on someone makes you fall in love. When you return to Bulgaria, it turns out that it’s not like that—there the environment and the harsh conditions make you.” This echoed her pre-trip understanding that distance from home would bind the team. Multiple participants used variations of the phrase, “there is no one else to turn to.” Another participant (Q4POST5) gave a vivid illustration: “If I do not rely on the mountaineer holding my rope over a crevasse, I could die—so even if I do not like him personally, I trust him with my life, and he trusts me with his.” This literal life-and-death interdependence fostered what participants called a “culture of helping.” The observer wrote that it was *“unthinkable”* at the base for someone not to offer help when another was in need. Veteran members had anticipated this all-hands approach, and no team member reported refusing to assist when asked. If anything, a few noted (with pride) that people sometimes *competed* to volunteer for extra chores or risky assignments—eager to pull their weight and demonstrate commitment to the group. Participant accounts and our field observations suggest that such mutual dependence was closely associated with stronger cohesion, consistent with prior research on polar environments (Palinkas, 2003; Suedfeld and Steel, 2000).

#### Leadership and “one team” culture

Leadership appeared to play an important role in team integration, as suggested by participants’ accounts and observer notes. Before deployment, participants identified him as “the person who has to synchronise the different teams and smooth out friction” (Q2PRE11). During the expedition, he organised all-hands meetings and rotated mixed crews, deliberately exposing different groups to one another. His informal yet authoritative style—willing to enforce group norms (e.g., instituting “dry” days during periods of tension)—reflects best practices for isolated crews (Palinkas, 2003; Palinkas and Suedfeld, 2008). His dual capacity as both respected leader and approachable colleague proved essential to cohesion.

#### Identity

The formation of a cohesive group was reflected in the evolution of the team’s group identity. Early on, identity was somewhat split by roles or pre-existing friendships (e.g., “the builders,” “the scientists,” “the newcomers” vs. “the old hands”). Nevertheless, as challenges were overcome collectively, a superordinate identity as “the Expedition team” became dominant. The observer’s notes describe how specific events (celebrating New Year’s together on the ice, jointly overcoming a dangerous situation involving a stuck boat, etc.) served as symbolic milestones that knit the group. These shared experiences created a narrative of “what *we* went through together,” reinforcing a common identity. By the latter half of the expedition, participants spoke of the team in collective terms far more often than in subgroups. This finding resonates with anthropological analyses that Antarctic stations develop their own microculture and group identity over time (Schweitzer, 2017; O’Reilly, 2017). In our case, the Bulgarian team took pride in being part of the national programme, but also developed its own microculture of inside jokes, routines, and norms that were unique to that season’s crew.

In summary, cohesion and community emerged strongly and were actively cultivated. Shared mission and mutual reliance were the foundation, leadership smoothed the process, and what started as a collection of different teams ended as a tight-knit community. A notable feature of our data is the centrality of national symbolism and pride in participants’ narratives—for example, describing themselves as ‘Bulgarian Antarctic explorers’, emphasising that ‘years from now we will tell our grandchildren we poured the concrete for the Antarctic base’, and reporting boosts in morale when others recognised their national mission during transit. While national symbolism has been noted in other polar programmes, the frequency and intensity of such references here suggest that, for this team, national identity functioned as an important psychosocial resource for cohesion and meaning-making, and this may reflect features of a less frequently studied Central/Eastern European national programme. At the same time, it may have encouraged socially desirable or positively framed accounts of the expedition, which we acknowledge as part of the study’s contextual limitations.

### Motivation trend and coping strategies

A central question for expedition members was how they would maintain motivation throughout the mission. Many had expressed confidence before departure that their enthusiasm would remain high, though some acknowledged potential psychological hurdles such as monotony, adverse weather, or personal lows. The post-mission accounts allowed us to trace a rough motivation timeline through the expedition and to identify key coping mechanisms the individuals and the group used to sustain morale. Overall, participants reported that their motivation remained surprisingly robust and, in several cases, even increased as the expedition progressed, aligning with their generally optimistic expectations.

In the following, we chart the team’s motivational trend over time by reporting motivational states at the start, midpoint, and end of the expedition. We then outline the coping mechanisms that shaped its trajectory.

### Motivation trend

Motivation peaked before departure, fueled by the unique opportunity and personal ambitions. Participants expressed dominant excitement despite some anxiety: “I was over 100% motivated—could not wait to get going” (Q3PRE4). Similar sentiments included ‘It must be at the maximum level of our motivation… we are highly motivated’—could not wait to get going’ (Q2PRE 3; 5; 13; 18; 20; 29).

In the first 2–3 weeks, motivation remained high but dipped slightly from exhaustion after the journey and demanding setup amid adverse weather: “The initial stormy week felt like a punch in the gut—my excitement waned” (Q3POST8). As routines formed and challenges were overcome, morale rebounded by weeks 3–4, marking a “norming” stage where newcomers gained confidence with veteran support and the team “found its rhythm” (Q3Post8).

In the final weeks, motivation surged to high levels as focus turned to wrapping projects and departure prep: “We realise that we have a unique opportunity that is limited. We want to accomplish maximum things, and we are highly motivated*”* (Q3PRE26). This “sprint” reflected common expedition patterns (Palinkas and Suedfeld, 2008), driven by legacy-building, long hours, and home excitement despite fatigue: “And then to the very end, the motivation is raised, because we are about to go home” (Q3POST13).

### Coping strategies

Various coping strategies were employed to prevent motivational dips and mood decline, particularly midway through the expedition. Participant coping operated across three integrated levels: individual self-regulation, team-based emotion management, and leadership oversight. At the individual level, expeditioners maintained functioning amid chronic monotony and weather constraints through personal routines and micro goals (e.g., daily exercise, journaling, achievable targets). When environmental disruptions occurred (e.g., storms, delays), cognitive reframing helped to re-interpret setbacks as opportunities for rest or social reconnection, thereby reducing perceived uncontrollability. At the group level, the team sustained cohesion and affective tone through humour, running jokes, and micro celebrations (themed dinners), using shared levity to down-regulate stress and sustain morale. Peer encouragement and recognising small wins restored motivation after setbacks. At the leadership level, station leaders monitored psychosocial risk through informal morale checks and one-to-one conversations, proactively organising group activities to address emerging declines in motivation and cohesion. A complete list of all observed and reported coping strategies is available in Table 2.

**Table 2:** Coping strategies by implementation level.

Strategy	Level	Trigger	What it involved	Example/evidence
Maintaining personal routines and small goals	Individual	Ongoing; especially during monotony or bad weather	Daily exercise, journaling, setting small achievable targets to create normalcy and a sense of progress	“A daily exercise regimen, keeping a journal” helped maintain momentum
Cognitive reframing	Individual	Delays, storms, schedule disruptions	Recasting setbacks as opportunities for rest, indoor tasks, or social time	“If it was storming, I told myself: great, now I can edit my field notes and drink hot cocoa.”
Humour and team spirit (micro-celebrations)	Group/team	Tough days; high stress	Running jokes, nicknames, themed dinners, small celebrations to release tension	“Someone would inevitably lighten the mood with a joke… laughter was a familiar sound even in stormy weather.”
Peer encouragement and shared emotional journey	Group/team	Low-motivation moments; after challenges	Pep talks, public recognition of wins, openly commiserating and celebrating together	“They would pat you on the back and say ‘we got this’… That kind of support was huge.”
Leadership monitoring and intervention	Leadership	Signs of flagging morale	Informal morale checks, one-on-one chats, organising group activities to preempt issues	Station leader and doctor “checked individuals’ morale” and initiated activities when needed

## Discussion

This study examined the human dimensions of an Antarctic research expedition through qualitative longitudinal analysis. Across interview waves and field notes, participants’ narratives depict a team that adapted and functioned effectively under demanding conditions, with many post-expedition accounts retrospectively framing the expedition as a high-performing unit. While we did not assess within-person dynamics, group-level patterns show that the participants’ initial motivation and positive expectations regarding teamwork and personal growth were validated throughout the expedition.

Participants’ narratives suggest that team cohesion increased markedly, progressing from sub-groups to a unified ‘community’ consistent with small-group development theory (Tuckman, 1965). Although minor “storming” occurred (e.g., logistics–science divisions), its resolution reinforced that silos should be actively countered through inclusive communication (Marques-Quinteiro et al., 2024; Palinkas, 2003). The expedition leader displayed several features of transformational leadership by balancing authority with approachability while managing fatigue and conflicts (Palinkas, 2003; Palinkas and Suedfeld, 2008). A shared identity and culture (exemplified by embedding national symbols and expedition rituals) strengthened cohesion (Tin et al., 2009; O’Reilly, 2017; Tajfel and Turner, 1979). Participants commonly described sub-team identities as having been subsumed by a superordinate ‘station team’ identity, and several explicitly linked this process to the positive effects of national symbolism.

Our thematic synthesis suggests a typical pattern at the group level—initial excitement, mid-mission variability with mild third-quarter dips, followed by renewed engagement—that aligns with polar and space-analogue research (Bechtel and Berning, 1991; Palinkas and Suedfeld, 2008; Hawkes and Norris, 2017). The mild third-quarter effect appeared to be mitigated, in participants’ accounts, by proactive stress buffering through structure, humour, and meaning-focused reframing (Suedfeld, 1991; Palinkas, 2003). Notably, participants reported less stress on-site than at home, supporting eustress models under purposeful challenge (Selye, 1974; Suedfeld and Steel, 2000). These patterns challenge a simple ‘inevitable deterioration’ model and are consistent with the possibility that, under appropriate conditions, individuals may maintain or even improve functioning throughout isolated expeditions. Rather than a universal third-quarter decline, mission-specific drivers (particularly tempo and meaningful milestones) emerged as key motivators (Hawkes and Norris, 2017).

Despite the interesting findings, our study has several important limitations. First, it examines a single national programme’s summer deployment with a particular team composition. Hence, the patterns we describe may be quite specific and may not generalise to other stations, seasons, or national programmes. Second, while our pre- and post-expedition design allowed us to contrast anticipatory expectations with retrospective narratives, some pre-expedition interviews were relatively brief (as short as five minutes), which might constrain the depth of comparison that reflexive thematic analysis can achieve for that wave. However, the shorter duration did not necessarily undermine their analytic contribution, particularly where experienced participants offered concise, focused accounts directly relevant to the study aims. Third, the anonymity protocol prevented linkage of individual pre- and post-expedition interviews; thus, our inferences concern aggregate, thematic change at the group level and cannot directly speak to strict within-person developmental trajectories or causal processes. Fourth, our data combine self-reports with the perspective of one embedded observer who was present for approximately 1 month of a 4.5-month campaign, which may introduce observational blind spots despite the triangulation matrix in Table 1. The observer’s 30-day window also means that their field notes could only partially complement self-reported motivation trends across the full 4.5-month campaign, so descriptions of mid- and late-mission motivation remain largely interview-based. Next, the high salience of shared national purpose and pride, which participants themselves frequently emphasised, raises the possibility of social desirability bias and positively framed retrospective narratives; we explicitly treat these as contextualised accounts rather than neutral descriptions of “what really happened.” Last, given our heavier reliance on longer post-expedition narratives for thematic illustration, some findings may also reflect post-hoc sense-making and reconstructed stories rather than contemporaneous experience. Future research could address these limitations through comparative, multi-team or multi-national designs, repeated quantitative measures, multiple observers covering a greater proportion of the mission, and more extensive pre-deployment interviews.

Several aspects warrant further investigation. As Antarctic programmes expand participation, understanding how gender and diversity dynamics influence team climate will become increasingly important (O’Reilly, 2017; Nash et al., 2019). The role of technology in mitigating isolation—particularly improved internet connectivity—represents another evolving area with psycho-social implications for Antarctic stations and other remote, high-risk settings such as offshore platforms or high-altitude research bases. Continued interdisciplinary research integrating psychology, human factors, and anthropology is essential to capture the full complexity of Antarctic exploration. Finally, by clarifying psychosocial factors that support effective and safe expedition functioning, our findings may inform planning not only for future polar missions, but also for other remote work settings characterised by environmental stressors, such as offshore oil and gas platforms, remote mining sites, high-altitude observatories, and desert field stations.

## Conclusion

Our account of Bulgaria’s 33rd Antarctic Summer Campaign illustrates that resilience in isolated, confined, and extreme settings can be understood not only as an individual trait but also as a team process: a group of specialists gradually becomes a cohesive micro-community through shared purpose, clear routines, and everyday mutual support. Participants’ accounts indicated that predictable stressors (e.g., monotony, weather disruption, early subgrouping) need not produce steady decline when identity and coordination are actively built over time.

For Antarctic research, our study supports process-based models of adaptation by showing how cohesion, leadership, and coping co-develop across a real mission cycle. For future expeditions, the implication is practical and optimistic: they can be designed for success by embedding early norm-setting, cross-role coordination, and reliable communication rituals into preparation and daily station life.

## Data Availability

The raw data supporting the conclusions of this article will be made available by the authors, without undue reservation.

## Footnotes

1 Q2PRE22; Q3PRE11/27; Q2POST14/22; Q3POST4; Q5POS4.
