# Supplementary material for: Leadership, adaptation, and group resilience: a qualitative study of Bulgaria’s 33rd Antarctic summer campaign
Source: Front Psychol. 2026 Apr 24;17:1820497. doi: 10.3389/fpsyg.2026.1820497 (PMC13152743; doi:10.3389/fpsyg.2026.1820497)
Supplement: Supplementary file 1 [file Table_1.docx]

Supplementary Material

Table A provides illustrative examples of deductive codes, inductive codes, and observer tags, together with the source material and the higher-order theme each code was grouped into.

| **Table A. Coding examples** | | | | |
| --- | --- | --- | --- | --- |
| **Code Type** | **Code Name** | **Source** | **Example from Study** | **Grouped Into Theme** |
| Deductive | Expected Unity Factors | Pre-expedition interview Q1 | P6 (Pre): "If we do not help each other, nothing will get done" | Cohesion & Community |
| Deductive | Experienced Unity Factors | Post-expedition interview Q1 | P1 (Post): "You have no one else to rely on but each other" | Cohesion & Community |
| Deductive | Motivation High Point | Pre-expedition interview Q2 | P2 (Post): "When we got the seismic station working, I was on a high for days" | Motivation Trajectories |
| Deductive | Motivation Dip | Post-expedition interview Q2 | P5 (Post): "Midway through, some days felt stuck on repeat" | Motivation Trajectories |
| Inductive | Leadership – Informal Style | Emerged from descriptions | Commander: "joked and worked alongside us" | Leadership & Cohesion |
| Inductive | Conflict Avoidance | Emerged from narratives | "resolved one-on-one" disputes | Leadership & Cohesion |
| Inductive | Personal Growth | Post-expedition reflections | P7 (Post): "Enhanced professional confidence, new skills" | Post-expedition Outcomes |
| Inductive | Group Cohesion | Multiple accounts | P10 (Post): "We became like a family – everyone depended on everyone" | Cohesion & Community |
| Observer Tag | Observation: Group Celebration | Field notes (transit/early) | Shared pride; National flag rituals | Cohesion & Community (Identity) |
| Observer Tag | Observation: Early Tension | Field notes (Week 1) | Scientist vs. logistics clustering; Newcomer vs. veteran divide | Inter-team Relations |
| Observer Tag | Observation: Norm Formation | Field notes (Weeks 2–4) | "Everyone finding their rhythm" by Week 3–4 | Norming Stage (Tuckman) |

Note: “Deductive” codes were specified a priori based on the interview guide and focal constructs; “Inductive” codes emerged during analysis; and “Observer tags” originate from field notes recorded by the embedded observer.
